# An Elastic Foot Orthosis for Limiting the Increase of Shear Modulus of Lower Leg Muscles after a Running Task: A Randomized Crossover Trial

**DOI:** 10.3390/ijerph192215212

**Published:** 2022-11-18

**Authors:** Kodai Sakamoto, Megumi Sasaki, Chie Tsujioka, Shintarou Kudo

**Affiliations:** 1 Inclusive Medical Science Research Institute, Morinomiya University of Medical Science, Osaka 559-8611, Japan; 2 Mikage Gokigen Clinic, Kobe 658-0048, Japan; 3 Yanase Orthopedic Clinic, Utsunomiya-shi 329-1115, Japan; 4 Kiba Hospital, Tokyo 135-0042, Japan; 5 Graduate School of Health Science, Morinomiya University of Medical Sciences, Osaka 559-8611, Japan; 6 AR-Ex Medical Research Center, Tokyo 158-0082, Japan

**Keywords:** foot orthosis, elastography, running, overuse injury

## Abstract

Background: Excessive foot pronation may be attributed to an increasing burden on leg muscles during running, which might be a factor in medial tibial stress syndrome. We developed an elastic foot orthosis (EFO) that can decrease foot pronation and aimed to identify whether this orthosis could limit the increase in lower leg muscle hardness after running. Methods: Twenty-one healthy volunteers participated in this randomized crossover trial with an elastic or sham foot orthosis (SFO). All volunteers ran on a treadmill for 60 min while wearing either orthosis. Muscle hardness of the posterior lower leg was assessed using shear wave elastography before and after running. The Wilcoxon signed rank test was used to compare muscle hardness between the two orthotic conditions. Results: No significant differences were observed between the two orthotic conditions before running (*p* > 0.05). After running, the flexor digitorum longus (FDL) hardness in the EFO group was significantly lower than that in the SFO group (*p* < 0.01). No significant changes were observed in the other muscles. Conclusion: The results suggest that the EFO can restrict the increase in FDL hardness with running. The EFO may be an effective orthotic treatment for medial tibial stress syndrome.

## 1. Introduction

Medial tibial stress syndrome (MTSS), commonly known as “shin splint” or “tibial periostitis”, is an overuse injury. It is characterized by exercise-induced pain along the distal two-thirds of the posteromedial surface of the tibia. Pain is usually elicited during sports activities or later [1,2]. In addition, studies on MTSS have shown that runners were restricted from participating in sports activities for over 9 weeks, with many cases of recurrence [3,4]. Clarifying the etiology and risk factors for MTSS is necessary to aid its prevention [5]. Although the etiology of MTSS is still debated, it has been hypothesized that the traction force of the lower leg flexor muscles (i.e., the soleus [SOL], tibialis posterior, or flexor digitorum longus [FDL]), could induce tibial periostitis [1]. Moreover, an excessively pronated foot is a risk factor for MTSS [6,7,8,9,10]. The FDL and tibialis posterior function as foot supinators, and their hardness may be increased in patients with an excessively pronated foot due to repeated elongation stress during running [11,12]. Controlled foot alignment during running may therefore help to limit the increase in the hardness of those muscles, thereby preventing both the onset and therefore treatment of MTSS.

Several approaches have been reported on controlling foot alignment. Kim and Park [13], and Bishop et al. [14] showed that sports taping could restrict the midfoot and medial longitudinal arch motion during walking and jogging. However, the effect of taping is dependent on the technique of application by medical staff or trainers. Foot orthoses are frequently prescribed for maintaining foot alignment and are not influenced by the treatment technique. Previous studies showed that foot orthoses could effectively control foot eversion and lower leg muscle activities [15,16]. In addition, Menéndez et al. [10] suggested that foot orthoses were effective in reducing the pain of patients with MTSS. However, most conventional foot orthoses function by being inserted into the sole of the individual shoe and are only suitable for well-fitting shoes. Moreover, although the kinematic effects of foot orthoses have been confirmed, few reports on foot orthoses that can limit the increase in hardness of the lower leg muscle with running exist.

We previously proposed an elastic foot orthosis (EFO) that supports the medial longitudinal arch during walking and running [17]. This EFO can be used together with various types of shoes because it is composed of thin films, which enables it to insert into footwear. However, its influence on lower leg muscle hardness has not been verified.

This study aimed to investigate the effect of the EFO on the increase in lower leg muscle hardness after a running task.

## 2. Materials and Methods

The protocol was approved by the Ethics Committee of the Morinomiya University of Medical Science (approval number: 2019−091). This study was registered in the University Hospital Medical Information Network Registry (UMIN000041314) and adhered to the CONSORT guidelines (see Supplementary Material Table S1).

### 2.1. Participants

Twenty-one healthy college students (8 men and 12 women, height, 170.0 ± 10.0 cm; weight, 61.0 ± 10.4 kg; age, 20.3 ± 1.3 years; foot length 24.7 ± 1.6 cm) who regularly performed recreational physical activities twice a week, participated in this study. None of the volunteers experienced lower limb pain before the study. Those who could not complete the running task or already wore an EFO were excluded. It used a randomized double-blind crossover design. We preliminarily conducted a calculation of sample size using G*Power 3.1 with power level and α level set at 80% and 0.5, respectively. The power analysis showed that 20 participants is sufficient to observe large differences between conditions.

### 2.2. Study Design

**Figure 1:**
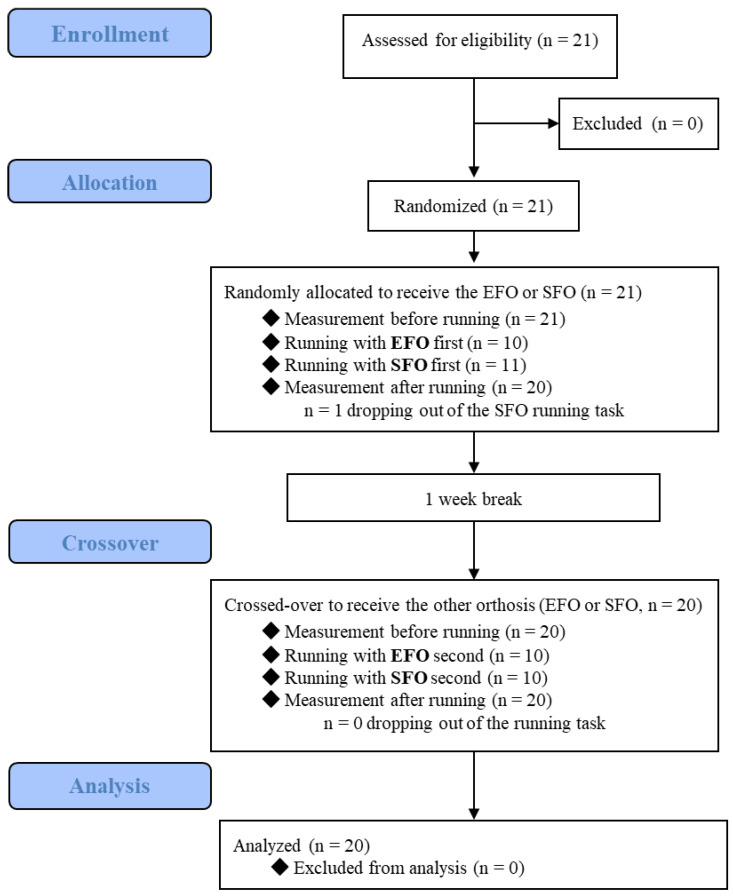
CONSORT diagram. EFO; elastic foot orthosis, SFO; sham foot orthosis.

The study procedure is summarized in Figure 1. We used a randomized double-blind crossover design. Participants were randomly assigned to one of two orthotic conditions using a random number table in a 1:1 allocation ratio: EFO or sham foot orthosis (SFO). Next, the muscle hardness was assessed by one examiner prior to a running task. Then, all subjects ran on the treadmill wearing either the EFO or SFO as assigned, at the first examination. A week after the first examination, the subjects performed the same running task with crossover to the other orthosis condition, and the same examiner evaluated the muscle hardness before and after, using the same measurement procedure.

### 2.3. Muscle Hardness Measurement

**Figure 2:**
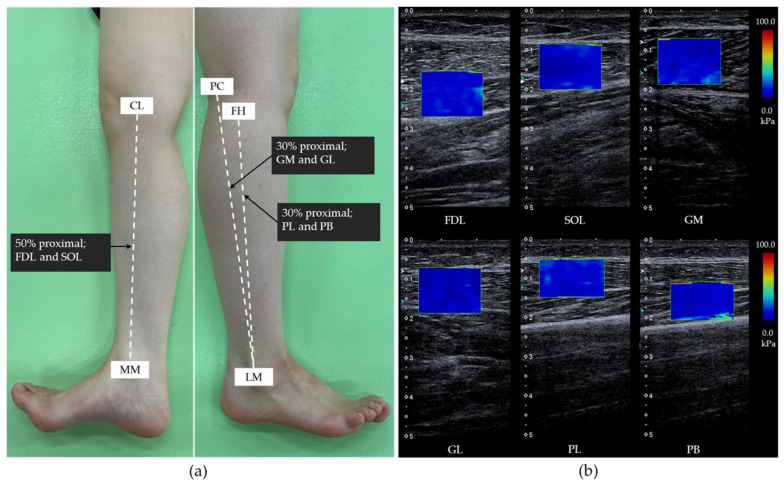
The measurement positions of the lower leg muscles (**a**). Elastographic ultrasound images (**b**). FDL: flexor digitorum longus; SOL: soleus; GM: gastrocnemius medialis; GL: gastrocnemius lateralis; PL: peroneus longus; PB: peroneus brevis. CL: cleavage line of the knee joint; FH: head of fibula; LM: lateral malleolus; MM: medial malleolus; PC: popliteal crease.

Muscle hardness was measured using ultrasonic shear wave elastography imaging (Aplio 500; Toshiba Medical Systems, Tokyo, Japan) and a 5–14 MHz linear probe. The shear elastic modulus in the long axis of the FDL, SOL, gastrocnemius medialis (GM), gastrocnemius lateralis (GL), peroneus longus (PL), and peroneus brevis (PB) were assessed as muscle hardness before and after the running task (Figure 2). The FDL, SOL, PL, and PB were measured with the subjects in a lateral position, and the GM and GL were evaluated while the subjects were in a prone position. During measurement, the subjects laid with the hip in the intermediate position, the knee in full extension, and the ankle in 0° dorsiflexion. The measurement locations of each muscle were determined by partially modifying them with reference to previous research [11,12]. FDL hardness was assessed at the midpoint of the line between the knee joint fissure and medial malleolus. The SOL hardness was obtained by moving the probe 1 cm backward from the FDL measurement position. The GM and GL hardness was measured at the proximal 30% of the lower leg length. PL and PB hardness were measured at the proximal 30% of the line joining the fibular head and lateral malleolus. Each measurement point was marked before the ultrasonic imaging procedure to place the probe at the same position before and after running. The quadrangular region of interest (ROI) was placed near the center of each muscle and was set to be as large as possible [11,12]. Subsequently, the elastic modulus of these muscles was measured at three random points within the ROI, and the mean value calculated. A pilot study to confirm the reliability of the shear elastic modulus measurements, with the same ultrasonic procedures, were repeated for nine healthy participants (5 men and 4 women, height, 164.1 ± 9.8 cm; weight, 64.7 ± 16.0 kg; age, 20.1 ± 0.3 years; foot length 23.5 ± 1.2 cm) without a running task over two days. 

### 2.4. Elastic Foot Orthosis

**Figure 3:**
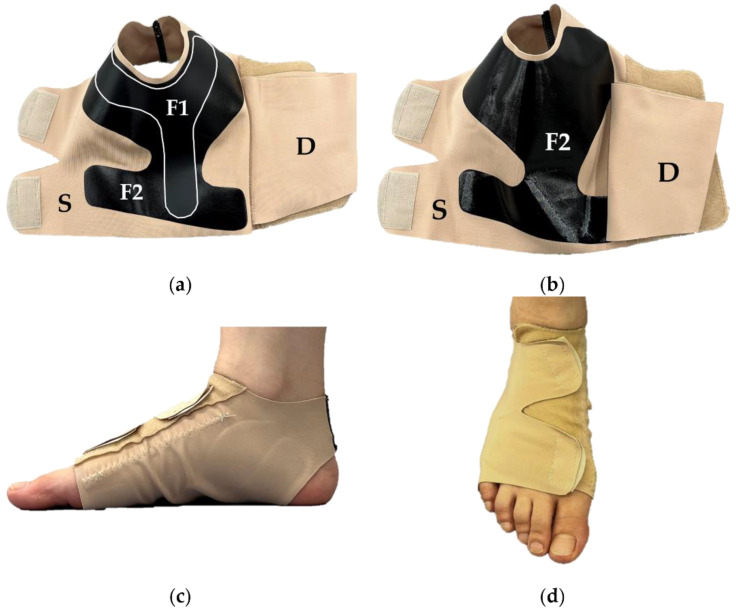
The pictures of the EFO and SFO. The EFO is composed of a deep part (D), superior part (S), and two types of thin hard films (F1 and F2) (**a**). The SFO was manufactured similarly to the EFO, but without F1 (**b**). The medial and the anterior views of the EFO (**c**,**d**). The orthoses are fastened using Velcro.

The EFO is comprised of four parts [17]. The superficial part wraps the entire foot, and the deep part covers the midfoot. These parts consist of a fabric that combines nylon and thermoplastic polyurethane. Two types of hard urethane films (F1 and F2) are inserted onto the plantar surface (both film thickness: 0.3 mm, density of urethane film: F1; 1.118 g/cm^3^, F2; 1.079 g/cm^3^). F1 was stiffer than F2 and was located from the midpoint between the first and second metatarsal heads through the inside of the sole to the heel. It covered the heel with bifurcating medial and lateral sides surrounding the medial calcaneus and cuboid bones. The SFO excluded the foot arch support mechanism present in the EFO. There were no great differences in appearance, feeling, and weight between the EFO and SFO, and participants were not told which was the EFO. Therefore, participants could not distinguish between these orthoses during examination. The structures of the EFO and SFO are illustrated in Figure 3.

### 2.5. Running Task and Measurement

Prior to the running task, one examiner measured muscle hardness. Subsequently, participants ran on a treadmill (Adventure 3 PLUS, Horizon, Johnson Health Tech Japan Co., Tokyo, Japan) at 4 km/h for a warm-up. The running speed was increased by 2 km/h every 90 s until it reached 8 km/h, at which point a 7% incline was applied, and the subjects continued the running task at the same speed for 60 min. This study included runners with any foot-strike pattern. All volunteers ran with the shoes they usually wore. If the participant could not continue running, or complained of pain, the running task was halted. The same examiner measured the hardness of each muscle again within 10 min after task completion. The examiner for the ultrasound measurements was blinded by being separated from the subject in a different room while participants ran and wore the EFO or SFO.

### 2.6. Statistical Analysis

Intraclass correlation coefficients (ICC) (1, 1) were used to evaluate the reliability of measuring the hardness of each muscle for the nine study participants. They were calculated based on the shear elastic modulus of the first and second days. The minimal detectable change (MDC) for muscles were calculated using the following formula:

MDC = standard error of measurement × √2 × 1.96,(1)

The difference in shear elastic modulus between participants with the EFO and SFO before running was assessed using the Wilcoxon signed-rank test. Similarly, the difference between the EFO and SFO groups were compared after running. Next, to compare before and after wearing the orthosis, the shear elastic modulus was compared between pre- and post-running using the Wilcoxon signed-rank test in the EFO and SFO conditions. Additionally, to compare sex differences in the effect of the EFO, the change in shear elastic modulus before and after running with the EFO was calculated and compared between men and women using the Mann–Whitney U-test. All statistical analyses were performed using SPSS statistical software version 25 (SPSS, IBM Co., Armonk, NY, USA). Data are described as median and interquartile range (IQR), and the alpha level for all tests was set at 0.05.

## 3. Results

### 3.1. Participants’ Characteristics

Nine participants were recruited for assessing the reliability of the ultrasound measurement. Twenty participants completed the running task, and one was unable to. The physical characteristics of the participants who completed the task are presented in Table 1.

### 3.2. Reliability of Ultrasound Measurements

The ICC for all muscles were >0.75, which were categorized as excellent according to the previous study [18]. The ICC and MDC values are listed in Table 2.

### 3.3. Comparison between EFO and SFO

Table 3 details the difference between the EFO and SFO before and after running, and pre-post changes in muscle hardness within each condition. Muscle hardness before running was not significantly different between the two orthotic conditions. A significant difference was observed in FDL hardness after running (*p* < 0.01), in which FDL hardness with the EFO (median, 18.3 kPa; IQR, 15.7–22.6 kPa) was lower than that with the SFO (median, 24.5 kPa; IQR, 19.9–29.8 kPa).

### 3.4. Comparison between Pre and Post Running Task

The FDL hardness after running with the SFO increased more than before running (*p* < 0.05). There were no other significant differences between before and after running in the SFO condition. In comparison, for the EFO condition, the LG, PL, and PB hardness were significantly increased after running (*p* < 0.05). No other significant differences were found in the EFO condition.

### 3.5. Sex Differences in the Effect of the EFO

The sex difference in the change in muscle hardness before and after running with the EFO is shown in Table 4. The change in the FDL hardness of women (median; 10.9 kPa, IQR; 2.8–31.9) was significantly greater than that for men (median; −9.5 kPa, IQR; −14.5 to −7.2) (*p* < 0.01).

## 4. Discussion

The purpose of the present study was to verify the ability of EFO to suppress the increase in hardness of the lower leg muscles during running. Although there was no difference before running, FDL hardness using the SFO significantly increased after running compared to using the EFO. Moreover, FDL hardness with the SFO significantly increased after running compared to before running with the SFO. However, no such change was observed with the EFO before and after running. Therefore, we found that the EFO was capable of limiting the increase in FDL hardness after running. On the other hand, LG, PB, and PL hardness in the EFO condition increased after running. However, there was no difference in hardness of these muscles after running between the two orthotic conditions. Thus, EFO and SFO differed in their impact on the FDL but not significantly on other muscles.

Previous studies have claimed that runners with MTSS or a history of MTSS have an increase in the hardness of the lower leg muscles after running, which might contribute to induction of its symptoms and recurrence [12,19]. Foot orthoses are frequently prescribed for patients with overuse injuries. Several biomechanical effects of foot orthoses have been reported, which indicate that support of the arch reduces the load on tissues while maintaining the medial longitudinal arch [15,20,21]. These findings imply that the increase in the hardness of muscles maintaining the arch could be inhibited by preventing the collapse of the arch. However, it was unclear if foot orthoses could control the increase in muscle hardness during running. The EFO used in the present study has been found to decrease the forefoot dorsiflexion and eversion motion with respect to the hindfoot, which is associated with collapse of the medial longitudinal arch [17]. Therefore, we believe the present study is the first to confirm that a foot orthosis capable of controlling the dynamic collapse of the foot arch can also prevent the increase in the hardness of the lower leg muscles during running.

Previous studies have reported that the collapse of the medial longitudinal arch may cause the development of MTSS [10]. The talonavicular joint is dorsiflexed and abducted with lowering of the medial longitudinal arch. Tsutsumi et al. suggested that the FDL and the tibialis posterior could contribute to the stability of the talonavicular joint, and the FDL tendon is stretched when loading [22]. The EFO may restrict the dorsiflexion and eversion of the forefoot with respect to the hindfoot [18]. As a result, the EFO may reduce the excessive elongation of the FDL during running and prevent the increase in FDL hardness. Furthermore, the results showed a greater effect of the EFO in men than women. It is known that the medial longitudinal arch in women tended to be lower than that in men [23,24]. Perhaps in women, a greater contribution of the FDL is necessary to support the foot arch, thus, the change in hardness of the FDL after running with the EFO was higher (i.e., the decreased hardness was reduced) compared to that in men.

The FDL attaches to the middle and distal thirds of the medial margin of the tibia [25], which is a frequent site of MTSS, and an increase in the strain of the FDL is attributed to the rising tension at that site through the tibial fascia [26]. These mechanisms suggest that excessively increasing FDL hardness could increase the traction force of the tibial periosteum, which may be related to the development and recurrence of MTSS. The EFO has the ability to prevent an increase in FDL hardness while running; therefore, it could be helpful in pain management and inhibition of recurrence of MTSS. In future studies, confirming the efficacy of EFO in prevention of MTSS is required.

This study has some limitations. First, the shoes used for the task varied amongst the participants. The construction or properties of shoes may influence muscle activity or kinematics during running. Verification of the EFO with standardized shoes is necessary. Next, we did not assess the tibialis posterior because this muscle is too deep to measure, with consistent reproducibility. The tibialis posterior supports the medial longitudinal arch similar to the FDL. Therefore, it is necessary to examine the influence of the EFO on this muscle in the future. Third, although the influence of EFO on gait was previously confirmed, this study did not confirm the correlation between the gait analysis and the change in muscle hardness. Verifying this correlation could provide a more detailed examination of the EFO effect on MTSS. Forth, this study did not consider differences in foot strike pattern, which might influence running kinematics. Fifth, the present study could not compare the effect of the EFO with other intervention types, such as insoles or taping. Comparisons with various interventions may be helpful to confirm the effectiveness of EFO clearly. Finally, the present study included only healthy subjects, and further studies should target patients with MTSS.

## 5. Conclusions

In conclusion, this study verified the effect of the EFO, which supports the foot arch and limits the increase in the hardness of the lower leg muscles during running. The EFO may be an effective orthotic treatment for MTSS, although further studies are required to demonstrate this.

## Figures and Tables

**Table 1:** Participant physical characteristics.

	*n*, Mean ± S.D.	
	Pilot Study	Randomized Cross Over Trial
Numbers (men/women)	9 (5/4)	20 (8/12)
height (cm)	164.1 ± 9.8	170.0 ± 10.0
weight (kg)	64.7 ± 16.0	61.0 ± 10.4
age (years)	20.1 ± 0.3	20.3 ± 1.3
foot length (cm)	25.5 ± 1.2	24.7 ± 1.6

**Table 2:** Reliability of muscle hardness measurements assessed in the pilot study with nine participants.

	ICC (1, 1)	ICC 95% CI	SEM	MDC
FDL	0.98	0.93–1.00	0.93	2.57
SOL	0.77	0.31–0.94	0.80	2.21
GM	0.97	0.87–0.99	1.96	5.43
GL	0.92	0.71–0.98	1.15	3.20
PL	0.82	0.43–0.96	2.98	8.27
PB	0.80	0.38–0.95	1.72	4.77

FDL, flexor digitorum longus; GM, gastrocnemius medialis; GL, gastrocnemius lateralis; ICC, intraclass correlation coefficient; MDC, minimal detectable change; PL, peroneus longus; PB, peroneus brevis; SOL, soleus.

**Table 3:** The hardness (kPa) of lower leg muscles between the two orthotic conditions before and after running.

	EFO		SFO		EFO vs. SFO Comparison		Pre vs. Post Change	
	Pre-Running Median (IQR)	Post-Running Median (IQR)	Pre-Running Median (IQR)	Post-Running Median (IQR)	Pre-Running *p*-Value	Post-Running *p*-Value	EFO *p*-Value	SFO *p*-Value
FDL	17.2 (14.1 to 20.6)	18.3 (15.7 to 22.6)	16.5 (13.9 to 21.5)	24.5 (19.9 to 29.8)	0.65	0.02	0.17	<0.01
SOL	13.1 (10.9 to 15.9)	14.4 (13.1 to 16.9)	13.8 (11.7 to 15.2)	15.3 (11.9 to 17.2)	0.97	0.88	0.10	0.24
GM	22.0 (17.1 to 25.6)	26.7 (22.1 to 29.3)	23.9 (21.6 to 28.0)	26.3 (22.8 to 34.4)	0.25	0.50	0.25	0.06
GL	19.9 (16.8 to 22.1)	25.9 (23.7 to 29.8)	21.8 (20.2 to 27.5)	22.8 (20.8 to 30.1)	0.07	0.55	0.01	0.32
PL	13.4 (11.3 to 16.1)	17.4 (12.7 to 23.9)	14.9 (11.2 to 18.9)	15.2 (12.9 to 19.2)	0.35	0.22	0.01	0.42
PB	12.5 (11.0 to 14.3)	15.8 (11.9 to 20.6)	11.8 (10.0 to 14.2)	15.0 (11.9 to 16.8)	0.63	0.29	0.01	0.05

FDL, flexor digitorum longus; GM, gastrocnemius medialis; GL, gastrocnemius lateralis; ICC, intraclass correlation coefficient; MDC, minimal detectable change; PL, peroneus longus; PB, peroneus brevis; SOL, soleus.

**Table 4:** Comparison between the sex difference in the change in muscle hardness before and after running with the EFO.

	Male, Median (IQR)	Female, Median (IQR)	*p*-Value
FDL	−9.5 (−14.5 to −7.2)	10.9 (2.8 to 31.9)	<0.01
SOL	5.7 (−9.7 to 27.4)	9.4 (−1.3 to 34.1)	0.54
GM	16.3 (−34.5 to 55.0)	27.4 (1.8 to 58.8)	0.59
GL	48.1 (5.7 to 55.1)	8.0 (−5.2 to 48.8)	0.59
PL	21.5 (−2.0 to 73.7)	32.2 (1.8 to 47.8)	0.94
PB	24.3 (−17.2 to 81.2)	21.2 (−0.3 to 73.5)	0.59

FDL, flexor digitorum longus; GM, gastrocnemius medialis; GL, gastrocnemius lateralis; ICC, intraclass correlation coefficient; MDC, minimal detectable change; PL, peroneus longus; PB, peroneus brevis; SOL, soleus.

## Data Availability

All data from the study are presented in the manuscript.

## Supplementary Materials

The following supporting information can be downloaded at: https://www.mdpi.com/article/10.3390/ijerph192215212/s1, Table S1: CONSORT 2010 checklist.
